# *P. aeruginosa* SGNH Hydrolase-Like Proteins AlgJ and AlgX Have Similar Topology but Separate and Distinct Roles in Alginate Acetylation

**DOI:** 10.1371/journal.ppat.1004334

**Published:** 2014-08-28

**Authors:** Perrin Baker, Tyler Ricer, Patrick J. Moynihan, Elena N. Kitova, Marthe T. C. Walvoort, Dustin J. Little, John C. Whitney, Karen Dawson, Joel T. Weadge, Howard Robinson, Dennis E. Ohman, Jeroen D. C. Codée, John S. Klassen, Anthony J. Clarke, P. Lynne Howell

**Affiliations:** 1 Program in Molecular Structure and Function, Research Institute, The Hospital for Sick Children, Toronto, Ontario, Canada; 2 Department of Biochemistry, Faculty of Medicine, University of Toronto, Toronto, Ontario, Canada; 3 Department of Molecular and Cellular Biology, University of Guelph, Guelph, Ontario, Canada; 4 Alberta Glycomics Centre and Department of Chemistry, University of Alberta, Edmonton, Alberta, Canada; 5 Leiden Institute of Chemistry, Leiden University, Leiden, The Netherlands; 6 Photon Sciences Division, Brookhaven National Laboratory, Upton, New York, United States of America; 7 Department of Microbiology and Immunology, Virginia Commonwealth University Medical Center and McGuire Veterans Affairs Medical Center, Richmond, Virginia, United States of America; University of Washington, United States of America

## Abstract

The O-acetylation of polysaccharides is a common modification used by pathogenic organisms to protect against external forces. *Pseudomonas aeruginosa* secretes the anionic, O-acetylated exopolysaccharide alginate during chronic infection in the lungs of cystic fibrosis patients to form the major constituent of a protective biofilm matrix. Four proteins have been implicated in the O-acetylation of alginate, AlgIJF and AlgX. To probe the biological function of AlgJ, we determined its structure to 1.83 Å resolution. AlgJ is a SGNH hydrolase-like protein, which while structurally similar to the N-terminal domain of AlgX exhibits a distinctly different electrostatic surface potential. Consistent with other SGNH hydrolases, we identified a conserved catalytic triad composed of D190, H192 and S288 and demonstrated that AlgJ exhibits acetylesterase activity *in vitro*. Residues in the AlgJ signature motifs were found to form an extensive network of interactions that are critical for O-acetylation of alginate *in vivo*. Using two different electrospray ionization mass spectrometry (ESI-MS) assays we compared the abilities of AlgJ and AlgX to bind and acetylate alginate. Binding studies using defined length polymannuronic acid revealed that AlgJ exhibits either weak or no detectable polymer binding while AlgX binds polymannuronic acid specifically in a length-dependent manner. Additionally, AlgX was capable of utilizing the surrogate acetyl-donor 4-nitrophenyl acetate to catalyze the O-acetylation of polymannuronic acid. Our results, combined with previously published *in vivo* data, suggest that the annotated O-acetyltransferases AlgJ and AlgX have separate and distinct roles in O-acetylation. Our refined model for alginate acetylation places AlgX as the terminal acetlytransferase and provides a rationale for the variability in the number of proteins required for polysaccharide O-acetylation.

## Author Summary

Bacteria utilize many defense strategies to protect themselves against external forces. One mechanism used by the bacterium *Pseudomonas aeruginosa* is the production of the long sugar polymer alginate. The bacteria use this polymer to form a biofilm – a barrier to protect against antibiotics and the host immune response. During its biosynthesis alginate undergoes a chemical modification whereby acetate is added to the polymer. Acetylation of alginate is important as this modification makes the bacterial biofilm less susceptible to recognition and clearance by the host immune system. In this paper we present the atomic structure of AlgJ; one of four proteins required for O-acetylation of the polymer. AlgJ is structurally similar to AlgX, which we have shown previously is also required for alginate acetylation. To understand why both enzymes are required for O-acetylation we functionally characterized the proteins and found that although AlgJ exhibits acetylesterase activity – catalyzing the removal of acetyl groups from a surrogate substrate – it does not bind to short mannuornic acid polymers. In contrast, AlgX bound alginate in a length-dependent manner and was capable of transfering acetate from a surrogate substrate onto alginate. This has allowed us to not only understand how acetate is added to alginate, but increases our understanding of how acetate is added to other bacterial sugar polymers.

## Introduction

*Pseudomonas aeruginosa* is an opportunistic, Gram-negative pathogen that can cause acute and chronic infections. The bacterium is the dominant bacterial species in the lungs of cystic fibrosis (CF) patients and if left untreated, is the leading cause of morbidity and mortality among these individuals [1]. *P. aeruginosa* is able to persist through the formation of a biofilm where communities of surface attached bacteria are encapsulated in a matrix composed primarily of secreted extracellular polysaccharides. Bacteria embedded within a biofilm are more resistant than their planktonic counterparts to environmental stresses such as antibiotics and disinfectants, and are able to evade the defense mechanism(s) of the host [2]–[7]. *P. aeruginosa* has the genetic capability to produce at least three different biofilm exopolysaccharides: Pel, Psl, and alginate [8], [9]. *P. aeruginosa* clinical isolates obtained from CF patients with chronic pulmonary infections secrete large amounts of alginate [10], [11]. This exopolysaccharide is synthesized in the cytoplasm and is translocated across the inner membrane as a linear homopolymer of d-mannuronic acid [12], [13]. The polymer is subsequently modified in the periplasm through O-acetylation and epimerization to form a β1–4 linked non-repeating chain of d-mannuronic acid and its C5 epimer l-guluronic acid [14], [15].

Modification of polysaccharides through the addition or removal of acetate is an important biological process for survival and virulence in many bacterial species. For example, biofilm formation by the human pathogens *Escherichia coli*, *Staphylococcus aureus* and *Staphylococcus epidermidis*, requires the partial de-*N*-acetylation of the exopolysaccharide poly-β-1,6-*N*-acetyl-d-glucosamine (PNAG) [16]–[19]. Similarly, deacetylation of Pel from *P. aeruginosa* is required for Pel-dependent biofilm formation [20] while deacetylation of the holdfast polysaccharide synthesized by *Caulobacter crescentus* is required for adhesion and cohesion [21]. In each case the deacetylation or removal of acetate from the acetylated polysaccharide requires a single enzyme, which has been shown to be a member of carbohydrate esterase family 4 (CE4). In comparison, O-acetylation of polysaccharides is a complex process requiring two enzyme functionalities. The first functionality is the transfer of an acetyl-donor into the periplasm, which is hypothesized to be catalyzed by a membrane bound O-acetyltransferase (MBOAT) [22]. The second functionality, catalyzed by a periplasmic O-acetyltransferase, transfers acetate onto the polysaccharide. There are currently three distinct systems that are differentiated by the number of proteins required for O-acetylation; a single protein system whereby both functionalities are encoded on one polypeptide, a two-protein system with one MBOAT and one periplasmic O-acetyltransferase and, in the case of alginate and cellulose, a four protein system comprised of an MBOAT protein, two periplasmic O-acetyltransferase and a protein of unknown function [22], [23].

The O-acetylation of the C6 hydroxyl of muramoyl residues in peptidoglycan is critical in many human bacterial pathogens including; Methicillin-resistant *S. aureus* (MSRA), *Bacillus anthracis*, *Neisseria meningitides* and *Neisseria gonorrhoeae* [24], [25] as it confers resistance to degradation by endogenous autolysins and the host immune system during infection [26]. A single integral membrane O-acetylpeptidoglycan transferase (Oat) is utilized in Gram-positive bacteria, while Gram-negative bacteria utilize a two-protein system composed of PatA, the MBOAT, and PatB, the periplasmic O-acetyltransferase [27]–[29]. The O-acetylation of alginate in *P. aeruginosa*, is an important modification as acetylated alginate is less susceptible to recognition and clearance by the host immune system than its non-O-acetylated counterpart [30]. Similar to the acetylation of cellulose by *Pseudomonas fluorescens* through the combined action of WssG, WssH, WssI and WssF, the O-acetylation of alginate requires four proteins; AlgF, AlgI, AlgJ and AlgX [12], [13], [31]–[33]. Acetylation of alginate can occur at the C2 and C3 hydroxyl groups of mannuronic acid residues. AlgI is predicted to be a member of the MBOAT family, while AlgJ and AlgX are required in the O-acetylation of alginate as the polymer passages through the periplasm [13], [31], [33]–[35]. The function of AlgF, which is not predicted to have a catalytic domain, is currently unknown. It is also unclear why alginate acetylation requires two active O-acetyltransferases.

To probe the role of AlgX in alginate acetylation, we recently determined its structure and found that it is a two-domain protein with an N-terminal SGNH hydrolase-like domain and a C-terminal carbohydrate-binding module (CBM) [34]. Our *in vivo* functional characterization of AlgX demonstrated that three catalytic residues, D174, H176 and S269, located in the active site are required for alginate O-acetylation. In the present study, we sought to delineate the role of AlgJ, and examine why both AlgJ and AlgX are required for alginate acetylation [13]. To this end, we have determined the structure of *Pseudomonas putida* AlgJ_75–370_ (*Pp*AlgJ_75–370_) to 1.83 Å resolution and have functionally characterized the protein.

## Results

### AlgJ contains an SGNH hydrolase-like core

The TMHMM server v2.0 indicates that AlgJ from *P. aeruginosa* PAO1 possesses a transmembrane helix from residues 12–29 that tethers the periplasmic domain to the cytoplasmic membrane [36]; a prediction that is supported by *in vivo* localization studies [13]. To probe the function of AlgJ in alginate biosynthesis, we attempted to crystallize a soluble domain, residues 79–379, of *P. aeruginosa* AlgJ (*Pa*AlgJ_79–379_). Although this protein was recalcitrant to crystallization, we were able to crystallize the orthologous domain from *P. putida*, *Pp*AlgJ_75–370_, which shares 83% and 53% sequence similarity and identity to *Pa*AlgJ_79–379_, respectively. Selenomethionine-incorporated (SeMet) protein was expressed, purified, crystallized, and the structure determined to 1.83 Å using the single-wavelength anomalous dispersion (SAD) technique (Table 1). *Pp*AlgJ_75–370_ crystallized in space group *C*2 with two molecules in the asymmetric unit. After iterative rounds of model building and refinement, the structure yielded models with an *R*_work_ and *R*_free_ of 17.5% and 21.2%, respectively (Table 1). Analytical size exclusion chromatography suggests that AlgJ is a monomer in solution (data not shown). The dimer observed in the crystal structure is therefore not believed to be biologically relevant. The two molecules in the asymmetric unit superimpose well with a root mean square deviations (RMSD) of 0.19 Å over the 906 aligned backbone atoms. Due to the poor quality of the electron density we were unable to build residues N75 to G77 and A266 to K278 in both molecules, and residues R78 and L242 in molecule A, and P244 and L245 in molecule B. Given the absence of two residues in the loop between L242 and F246 in molecule B, all structural analyses were performed using molecule A. All structural features defined using molecule A were also present in molecule B with no significant deviations.

**Table 1:** Summary of data collection and refinement statistics.

**Data Collection Statistics**	
Beamline	NSLS X29
Wavelength (Å)	0.979
Temperature (K)	100
Space Group	*C*2
Cell dimensions	
*a, b, c* (Å)	192.5, 34.0, 82.9
*α, β, γ* (°)	90.0, 91.9, 90.0
Total no. of reflections	358026
No. of unique reflections	48465
Resolution (Å)	50.00 – 1.81 (1.90 – 1.81)^a^
Average *I*/*σI*	16.0 (2.9)
Completeness (%)	98.8 (96.2)
Redundancy	7.5 (5.8)
*R*_merge_ b	10.1 (56.8)
**Refinement Statistics**	
*R*_work_/*R*_free_ (%)^c^	17.6/21.5
No. atoms	
Protein	4482
Water	282
Average B-factor (Å^2^)	
Protein	39.4
Water	43.8
Rms deviations	
Bond lengths (Å)	1.41
Bond angles (°)	0.014
Ramachandran plot^d^	
Total favoured (%)	98.2
Total allowed (%)	1.8
Coordinate error (Å)^e^	0.16
PDB Code	4O8V

a Values in parentheses correspond to highest resolution shell.

b *R*_merge_ = Σ Σ |I(k)−<I>|/Σ I(k) where I(k) and <I> represent the intensity values of the individual measurements and the corresponding mean values. The summation is over all unique measurements.

c *R*_work_ = Σ ∥*F*obs|−k|*F*calc∥/|*F*obs| where *F*obs and *F*calc are the observed and calculated structure factors, respectively. *R*_free_ is the sum extended over a subset of reflections (4.2%) excluded from all stages of the refinement.

d As calculated using MOLPROBITY [82].

e Maximum-Likelihood based coordinate error as determined by PHENIX [64].

**Figure 1:**
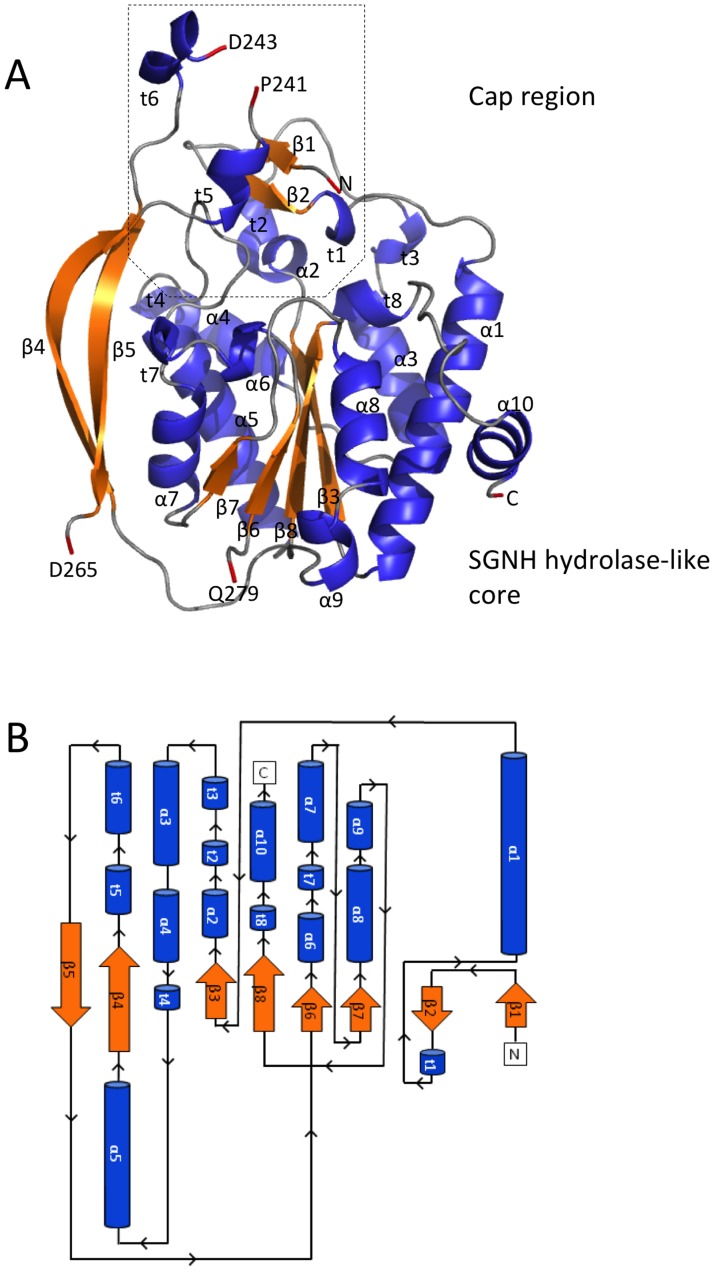
Structure and topology of *Pp*AlgJ_75–370_. (A) Cartoon representation of *Pp*AlgJ_75–370_ with secondary structural elements labelled (α: α-helix; β: β-strand; and t: 3_10_ helix). Residues at discontinuous points in the structure due to poor observed electron density are labelled and coloured red. The N- and C-termini of the protein are labelled N and C, respectively, and the terminal residue is coloured red. (B) Topology model of the *Pp*AlgJ_75–370_ structure with secondary structural elements and termini labelled as in panel (A).

**Figure 2:**
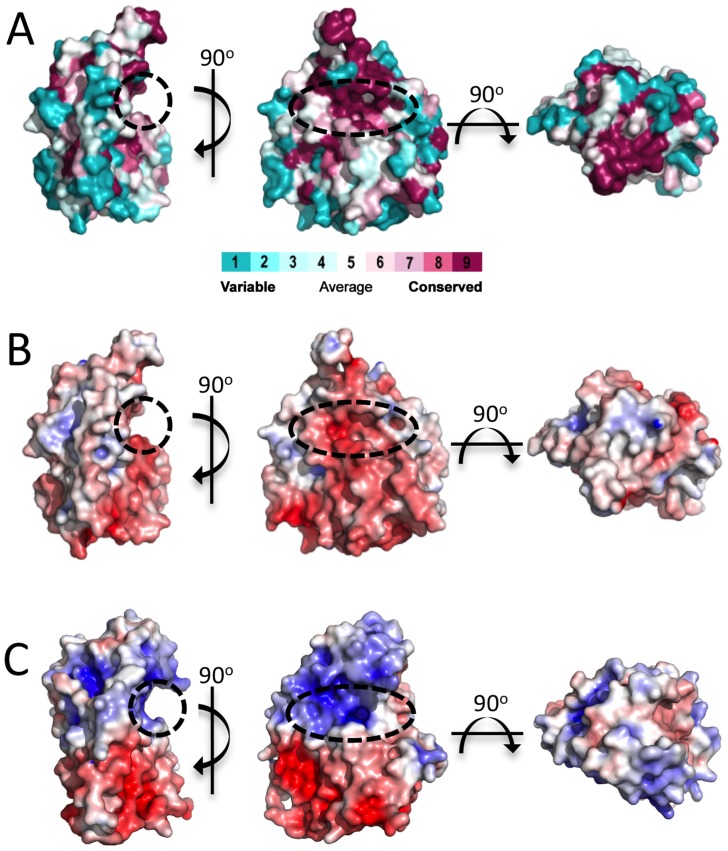
Surface representations of *Pp*AlgJ_75–370_ and its comparison to AlgX_44–344_. (A) Surface representation of *Pp*AlgJ_75–370_ with residue conservation of AlgJ homologs from 6 different *Pseudomonas* spp. and *A. vinelandii*. Conservation is displayed from magenta (highly conserved) to cyan (variable). Surface residue conservation was analyzed using ConSurf [71]. The dashed circle indicates the region where the shallow groove was identified. (B) and (C) Electrostatic surface representation of *Pp*AlgJ_75–370_ and *Pa*AlgX_44–344_, respectively. Electrostatic surface potentials were calculated using the APBS Tools 2.1 plugin within PyMOL [70]. The electrostatic surfaces are displayed in both panels from −7 kT/e (red) to +7 kT/e (blue).

The SGNH hydrolase superfamily consists of enzymes with varying hydrolytic activities (e.g., proteases and lipases) [37]. SGNH hydrolases have an α/β/α fold with each of the four conserved active site residues responsible for catalysis residing in one of four conserved blocks. The structure of *Pp*AlgJ_75–370_ reveals that the protein has an α/β/α fold with a core of four parallel β-strands (Figure 1: β3, β6–8) and an isolated β-bridge at β3, which are comparable to the five parallel β-strands found in canonical SGNH hydrolases [37], [38]. The core β-strands are surrounded by nine α-helices (Figure 1: α1, α3–10) that complete the α/β/α fold. A surface representation of *Pp*AlgJ_75–370_ reveals a shallow groove that crosses the face of the protein (Figure 2A, left, centre). Examination of the electrostatic surface potential shows a distinct region of electronegativity on the face of the protein located within and below the shallow groove (Figure 2B, centre).

Previous *in vivo* complementation experiments have suggested that AlgJ functions as an *O*-acetyltransferase and that this activity is dependent on conserved residues D193 and H195 (*P. aeruginosa* numbering) [33]. In *Pp*AlgJ the corresponding residues D190 and H192 are located in the shallow electronegative groove (Figure 2B, centre). Residues within the groove are well conserved in AlgJ homologs from six *Pseudomonas sp.* (*P. putida*, *P. aeruginosa*, *P. syringae*, *P. protegens*, *P. entomophila*, and *P. alkylphenolia*) and *Azobacter vinelandii* (Figure 2A, centre). A 90° rotation along the horizontal axis relative to the groove depicts a generally electroneutral surface (Figure 2B, right) that also contains highly conserved residues (Figure 2A, right).

**Figure 3:**
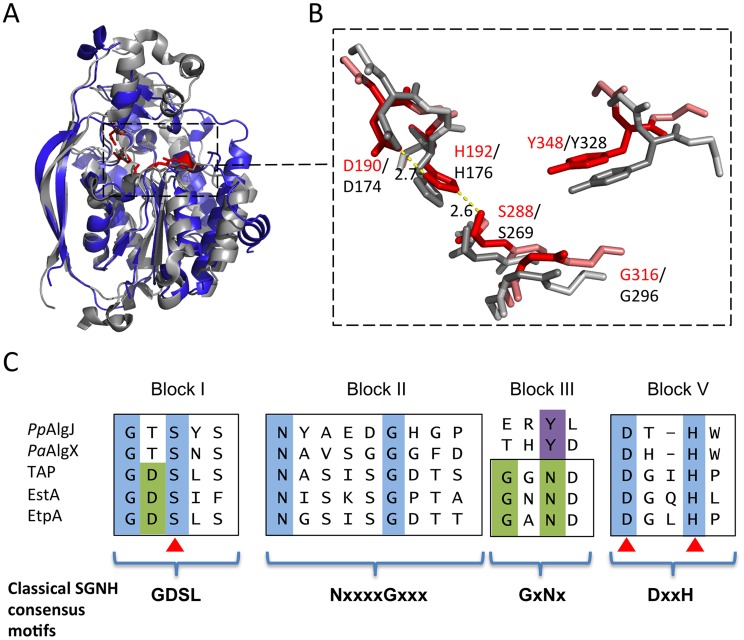
Architecture of the proposed *Pp*AlgJ_75–370_ active site compared to AlgX. (A) Superposition of the cartoon representations of *Pp*AlgJ_75–370_ (blue) and *Pa*AlgX_44–344_ (grey). The conserved residues implicated in catalysis in SGNH hydrolases are displayed as sticks and coloured either red (*Pp*AlgJ) or grey (AlgX). The dashed box indicates the region where the conserved residues are located. (B) Enlarged figure highlighting the similarity of the residues in the active site region in both proteins. Red stick residues and text identifies the conserved residues from *Pp*AlgJ whereas the grey sticks and black text identifies residues from AlgX. Yellow dashed lines represent hydrogen bonding between members of the catalytic triad with values in angstroms (Å). (C) Sequence alignment of various members of the SGNH hydrolase superfamily depicting four consensus blocks shared by members. *Pp*AlgJ: *P. aeruginosa* O-acetyltransferase (Q88ND3), PaAlgX: *P. aeruginosa O*-acetyltransferase (Q51372) EstA: *Lactobacillus helveticus* arylesterase (Q9LAH7), EtpA: *Vibrio mimicus* arylesterase (Q07792). Conserved residues are masked in blue (absolutely conserved) or green (conserved in most members). Residues masked in purple correspond to the conserved tyrosine in AlgJ and AlgX that are proposed to be analogous in function to asparagine in other superfamily members. Brackets correspond to Uniprot identifiers.

A search for structurally similar proteins using DALI reveals that the core of AlgJ is most similar to the N-terminal domain of *P. aeruginosa* AlgX, residues 44–344 (*Pa*AlgX_44–344_), with an RMSD of 2.06 Å over 165 aligned Cα-atoms (Figure 3A). The active site of *Pp*AlgJ_75–370_ and the orientation of the putative catalytic triad D190, H192 and S288, is analogous to *Pa*AlgX_27–474_ and other serine esterases and proteases (Figure 3B) [39], [40]. Similar to *Pa*AlgX_27–474_, the structure of *Pp*AlgJ_75–370_ reveals several differences relative to canonical SGNH hydrolases [32]. Firstly, block III that contains the conserved asparagine residue that forms part of the oxyanion hole is absent (Figure 3C). Examination of the *Pp*AlgJ_75–370_ structure suggests that Y348 is in a position to act as a hydrogen bond donor in place of the conserved asparagine (Figure 3B). In *Pa*AlgX_27–474_ a tyrosine residue, Y328, also occupies this position. *Pp*AlgJ_75–370_ also deviates from the canonical GDSL(S) and DxxH motifs in blocks I and V, respectively (Figure 3C). AlgJ has a GTSYS motif in block I which contains the catalytic serine (S288), while a single spacer residue in block V separates the two remaining catalytic triad residues (D190 and H192) to form a DxH motif. AlgJ appears to be a circularly permuted member of SGNH hydrolases as the order of conserved residues in primary sequence (H-S-G-Y) is different than that of SGNH hydrolases (S-G-N-H). However, the overall three dimensional shape and fold are structurally similar despite the rearrangement of conserved residues. *Pp*AlgJ_75–370_ also contains secondary structure features not present across SGNH superfamily members. Two long anti-parallel β-strands are present on one side of the protein (β4 and β5, Figure 1), along with eight 3_10_ helices (t1–8) and a small cap domain above the proposed active site which consists of two short anti-parallel β-strands (β1–2), five 3_10_ helices (t1–3, t5–6) and one α-helix (α2) (Figure 1). Despite these differences, *Pp*AlgJ_75–370_ is structurally comparable to other SGNH hydrolases including *E. coli* thioesterase I (TAP), and *Aspergillus aculeatus* Rha [38] as the SGNH hydrolase domains align with RMSDs of 3.2 Å and 3.8 Å over 101 and 104 equivalent Cα residues, respectively.

### The AlgJ signature motifs

**Figure 4:**
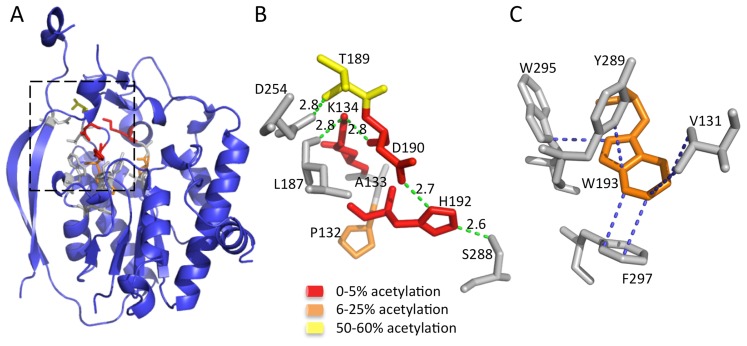
The AlgJ signature motifs. (A) Cartoon representation of *Pp*AlgJ_75–370_ with residues from the signature motif represented as sticks. The AlgJ signature motif residues have been coloured according to the amount of alginate acetylation observed *in vivo* in each alanine variant relative to WT AlgJ. Yellow represents impairment of between 50–60% acetylation; orange represents strong impairment with only 6–25% acetylation observed; and red represents ablation or between 0–5% observed acetylation. Residues represented as grey sticks are proposed to be involved in interactions with the AlgJ signature motif residues but have not been characterized *in vivo*. (B) Selected residues from the box area in panel A that participate in or are proposed to be involved in the hydrogen bonding network and are coloured as described in (A). Hydrogen-bonds are depicted as green dashes with distance given in angstroms (Å). (C) Residues participating in hydrophobic and van der Waals interactions with W193 (shown in orange) are depicted in grey. Hydrophobic and van der Waals interactions less than 3.7 Å are depicted with blue dashes.

Two conserved sequence motifs, termed the AlgJ signature motifs, have been defined in *P. aeruginosa* AlgJ and its homologs [33]. These motifs are characterized by conserved regions of ΦΦΦPxK (Φ represents any hydrophobic residue), and (R/K)TDTHW. Mutation of residues within these signature motifs leads to impairment or ablation of alginate O-acetylation [33]. Utilizing the structure of *Pp*AlgJ_75–370_, we identified the location of the signature motif residues (Figure 4A). Two distinct types of intramolecular interaction networks are observed within the motifs, which localize to the cap domain. The cap domain sits atop the SGNH hydrolase-like core that typically contains the active site and catalytic residues of canonical SGNH hydrolases [37], [38]. The first network composed of residues; K134, T189, D190 and H192 form a hydrogen-bonding network with L187, D254 and S288 (Figure 4B) in *Pp*AlgJ_75–370_. It was previously observed that alginate O-acetylation was abrogated *in vivo* for the *Pa*AlgJ variants K137A, D193A and H195A [33]. Superposition of *Pp*AlgJ_75–370_ with AlgX_44–344_ suggests that D190 and H192 (D193 and H195 in *Pa*AlgJ_79–379_) form part of the catalytic triad (Figure 3). Thus, two of the three catalytic triad residues (aspartic acid and histidine) in AlgJ reside in the separate cap domain distinct from the α/β/α fold, yet occupy an equivalent spatial position in the active site to their SGNH counterparts. The location of D193 and H195 in *Pa*AlgJ (D190 and H192 in *Pp*AlgJ) and the ablation of acetylation *in vivo* for the D193A and H195A variants provide further evidence of their crucial role in the catalytic mechanism of the protein. The second intramolecular interaction network is composed of a series of hydrophobic interactions centered around the conserved W193, with V131, Y289, W295 and F297 (Figure 4C). W193 is completely buried and comprises part of the hydrophobic core of AlgJ.

### The Ser-His-Asp triad is responsible for acetylesterase activity *in vitro*

**Figure 5:**
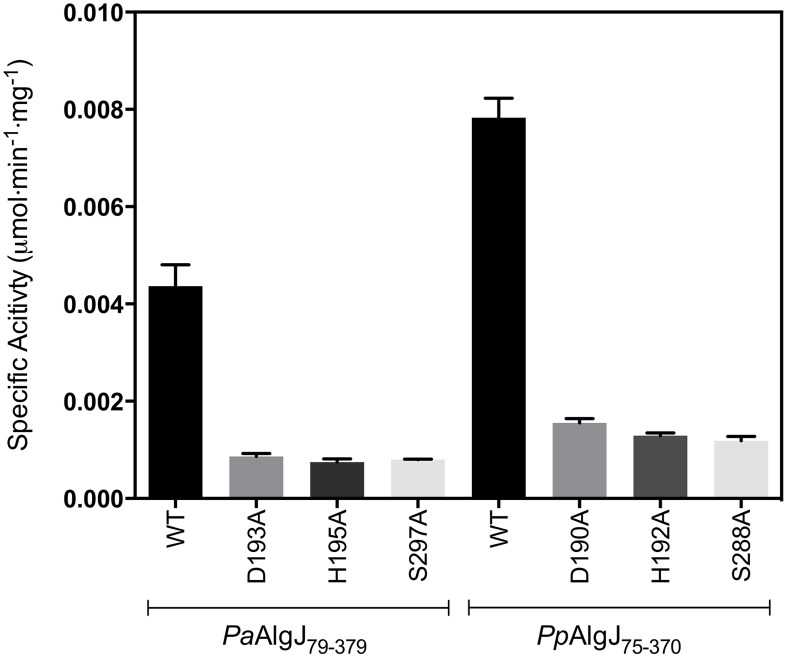
Specific activities of wild-type and catalytic triad mutants of *Pp*AlgJ_75–370_ and *Pa*AlgJ_79–379_. Specific activities were measured at an initial concentration of 3.0 mM ACC. WT and specific mutants for each AlgJ construct are labelled below the x-axis. Specific activity upon the y-axis was measured as µmol min^−1^ mg^−1^.

To determine whether AlgJ is catalytically active we examined the ability of the enzyme to exhibit *O*-acetylesterase activity, a commonality among SGNH hydrolases and the first half of the acetyltransferase reaction. *Pa*AlgJ_79–379_ and *Pp*AlgJ_75–370_ were both assayed to demonstrate that the enzymes are functionally equivalent. Using the substrate 3-carboxyumbelliferyl acetate, the kinetic parameters for *Pa*AlgJ_79–379_ and *Pp*AlgJ_75–370_ were observed to be comparable (Table 2), with a 2-fold difference in *K*_m_. The *k*_cat_/*K*_m_ obtained for AlgX, which has been previously demonstrated to catalyze this reaction, differed by only 3-fold compared to the AlgJ orthologs. To assess the function of putative catalytic residues; D190, H192 and S288 in *Pp*AlgJ and D193, H195 and S297 in *Pa*AlgJ were substituted with alanine. Interestingly, while catalytic alanine variants in AlgX result in the abrogation of 3-carboxyumbellifyl acetate hydrolysis [32], mutation of the catalytic triad in both AlgJ orthologs only reduced the catalytic activity by ∼80% (Figure 5). Circular dichroism spectroscopy of the AlgJ orthologs and their respective variants exhibited no significant difference in spectra (data not shown). This indicates that the protein variants are properly folded and that the differences in catalytic activity are not due to large structural perturbations.

**Table 2:** Summary of kinetic parameters^a^.

	*K*_m_ (mM)	*k*_cat_ (×10^3^ s^−1^)	*k*_cat_/*K*_m_ (M^−1^•s^−1^)
*Pa*AlgJ_79–379_	1.16±0.120	3.44±0.129	2.96±0.324
*Pp*AlgJ_75–370_	2.30±0.0963	7.80±0.155	3.39±0.0688
*Pa*AlgX_27–474_	1.10±0.0960	11.4±0.343	10.3±0.961

a Standard reactions contained variable concentrations of 3-carboxyumbelliferyl acetate (ACC), dissolved in DMSO ranging between 0.1 *K*_m_ and ≥2 *K*_m_ and 30 µg each protein in a total volume of 100 µL in 50 mM sodium HEPES buffer (pH 7.0 for AlgJ, pH 8.0 for AlgX) at 298 K.

### AlgX but not AlgJ interacts with mannuronic acid oligomers

**Figure 6:**
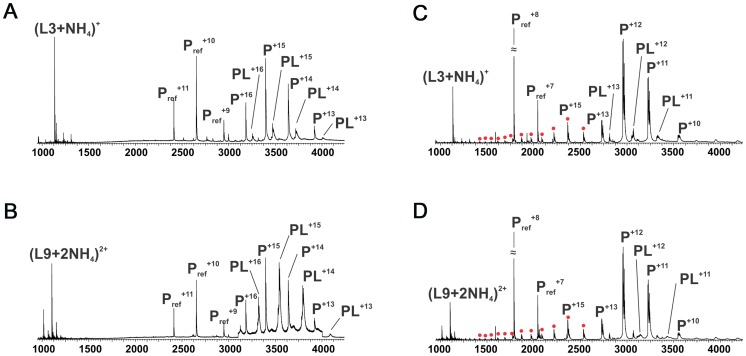
ESI mass spectra to examine polymannuronic acid binding. ESI mass spectra acquired for aqueous ammonium acetate (50 mM) solutions of (A) *Pa*AlgX_27–474_ (P) (4 µM) and 50 mM ManA_6_); (B) *Pa*AlgX_27–474_(P) (4 µM) and 50 mM ManA_12_); (C) *Pa*AlgJ_79–379_ (P) (10 µM) and 50 mM ManA_6_); (D) *Pa*AlgJ_79–379_ (P) (10 µM) and 50 mM ManA_12_). A reference protein scFv (4 µM) was added into the solutions of AlgX, Lyz was added as P_ref_ to AlgJ solutions. Ion peaks corresponding to higher charge states of AlgJ are labelled as filled red circles. P_ref_ refers to the reference protein scFv.

Comparison of *Pp*AlgJ_75–370_ and AlgX_44–344_ indicates a significant difference in electrostatic surface potential between the active site regions of the enzymes (Figure 2B and C). *Pp*AlgJ_75–370_ contains a shallow electronegative groove architecture around the active site whereas AlgX_44–344_ contains a deep electropositive groove compatible with binding the anionic alginate orpolymannuronic acid polymer. In addition, AlgX has a C-terminal carbohydrate-binding module, which presumably aids in binding and guiding alginate either to or from the active site. To examine whether *Pa*AlgJ_79–379_ and AlgX_27–474_ interact with mannuronic acid oligomers, a direct electrospray ionization mass spectrometry (ESI-MS) binding assay was carried out using nine mannuronic acid oligosaccharides ranging from 4–12 sugar units in length (ManA_4_ – ManA_12_). Representative ESI mass spectra acquired for aqueous ammonium acetate solutions of AlgX and the protein reference scFv (P_ref_) with ManA_6_ or ManA_12_ are shown in Figures 6A and 6B, respectively. In the mass spectrum shown in Figure 6A, ion signals corresponding to protonated AlgX monomer and protonated 1∶1 (AlgX+ManA_6_) complex, the +13 to +16 charge states, are observed. Signals corresponding to protonated P_ref_ and (P_ref_+ManA_6_ or ManA_12_) are also present, indicating that nonspecific carbohydrate-protein binding took place during the ESI process. The mass spectrum shown in Figure 6B is qualitatively similar to that shown in Figure 6A, although a visibly larger fraction of the protein is in the bound form. Similar results were obtained for the other alginate ligands tested (data not shown). ESI-MS measurements were also performed on solutions of AlgX and undeca- and pentadeca-hyaluronic acid (HA_11_ and HA_15_). Importantly, these negative controls revealed no evidence of specific binding between AlgX and these acidic oligosaccharides, thereby confirming the specificity of AlgX for the alginate oligomers rather than any acidic oligosaccharide. Listed in Table 3 are the association constants (*K*_a_) determined by ESI-MS, following correction for nonspecific carbohydrate-protein binding. Notably, the *K*_a_ values for AlgX are seen to clearly increase with the length of the mannuronic acid oligosaccharides. It must be noted that the location of ligand binding site cannot be explicitly identified using this methodology and the observed interaction for AlgX may include both ligand-active site and ligand-CBM interactions.

**Table 3:** Apparent association constants (*K*_a_) for *Pa*AlgX_27–474_ and *Pa*AlgJ_79–379_ for short polymannuronic oligosaccharides at 298 K and pH 7 determined by the direct ESI-MS assay.

Length of oligosaccharide (Ligand name)*	Apparent *K*_a_ (M^−1^) AlgX	Apparent *K*_a_ (M^−1^) AlgJ
4 (ManA_4_)	(1.0±0.5)×10^3^	<500
5 (ManA_5_)	(1.0±0.5)×10^3^	<500
6 (ManA_6_)	(3.2±0.3)×10^3^	<500
7 (ManA_7_)	(4.8±1.0)×10^3^	<500
8 (ManA_8_)	(5.8±1.0)×10^3^	NB
9 (ManA_9_)	(8.8±1.0)×10^3^	NB
10 (ManA_10_)	(10.0±0.7)×10^3^	NB
11(ManA_11_)	(16.0±0.5)×10^3^	NB
12 (ManA_12_)	(19.0±0.3)×10^3^	NB

NB: No Binding.

*Ligand name as referenced in [78].

The same assay was utilized to quantify binding of *Pa*AlgJ_79–379_ to the mannuronic acid oligomers. Representative ESI mass spectra are shown in Figures 6C and D for ManA_6_ and ManA_12_, respectively. Analysis of the ESI mass spectra reveals two distinct charge state distributions (+10 to +13 and +14 to +26) for the protonated ions of AlgJ. Ions corresponding to 1∶1 complexes of AlgJ with alginate ligand were only observed at the lower charge states. Taken together, these results suggest that a fraction of AlgJ is unfolded in solution (corresponds to the +14 to +26 charge state distribution) and does not bind to the oligosaccharides [41]–[45]. Only the lower charge state AlgJ ions were considered for the *K*_a_ determinations (Table 3). Because the relative protein ion abundances measured by ESI-MS do not necessarily reflect relative concentrations in solution, the concentration of folded AlgJ used for the *K*_a_ calculations was not accurately known. Nevertheless, the ESI-MS results suggest that under the conditions tested binding of AlgJ to the mannuronic acid oligomers is extremely weak, with *K*_a_ values of less than 500 M^−1^.

### AlgX is able to O-acetylate alginate *in vitro*

**Figure 7:**
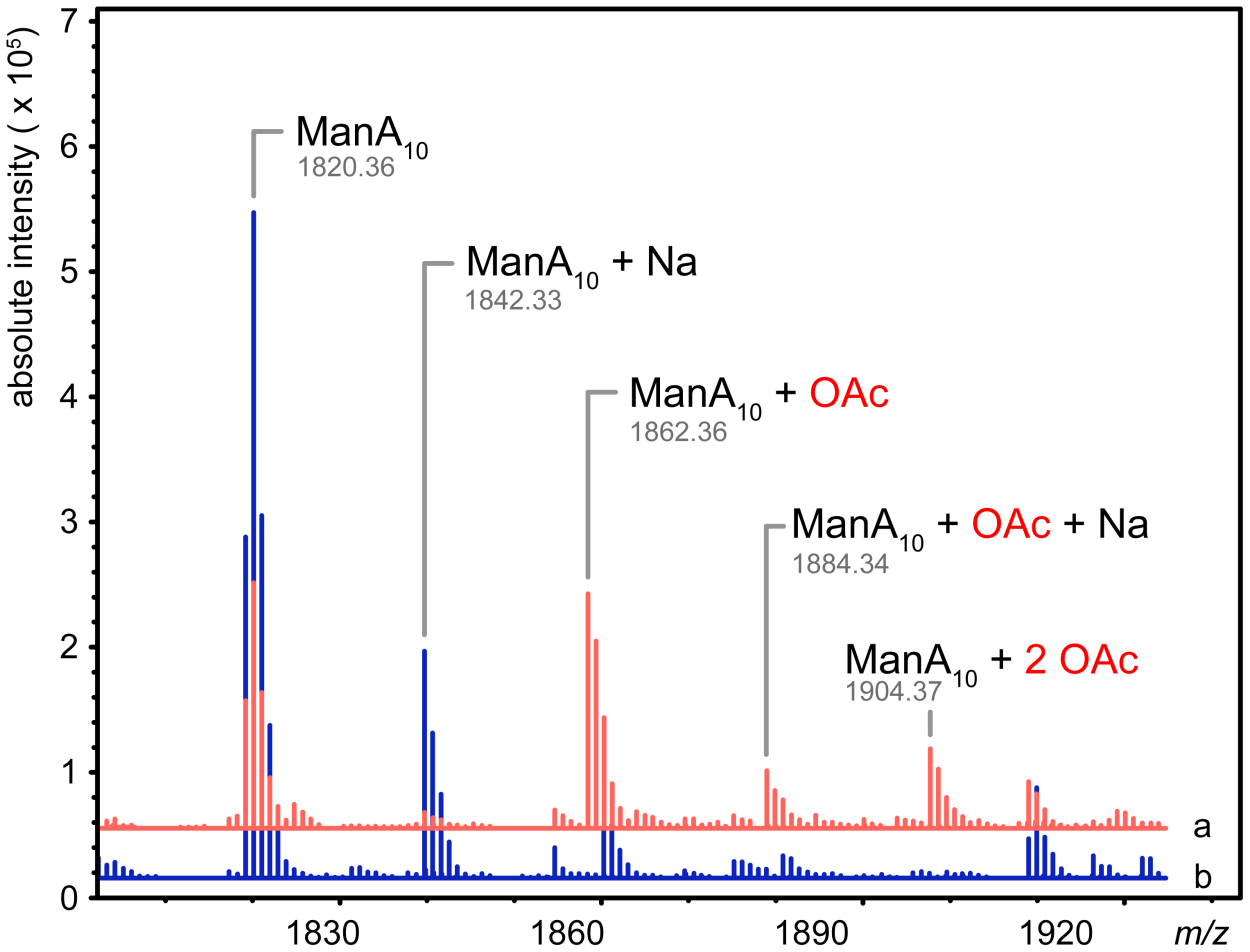
AlgX_27–474_ is an *O*-acetyltransferase. Mass spectra of the acetyltransferase reactions in the presence (red, line a) and absence (blue, line b) of *Pa*AlgX_27–474_. Major peaks are labelled: ManA10, mannuronic acid decamer; ManA10+Na, mannuronic acid decamer plus sodium; ManA10+OAc, mannuronic acid decamer plus acetate; ManA10+Na+OAc, mannuronic acid decamer plus sodium and acetate; ManA10+2 OAc, mannuronic acid decamer plus two acetates. The observed molecular weights of each product are denoted under their respective labels. The addition of acetate adds 42.01 Da to the mass of the polymer. The data has been deconvoluted and centroided using Agilent MassHunter software and represents uncharged exact masses.

Since AlgX interacts with mannuronic acid oligomers under the conditions tested, we examined the ability of AlgX to transfer an acetyl group from the pseudo-acetyl donors, 4-nitrophenyl acetate and 3-carboxyumbelliferyl acetate, to a decamer of polymannuronic acid (ManA_10_). Reactions analyzed by ESI-MS revealed the production of both mono- and di-acetylated mannuronic acid oligomers in the AlgX containing reactions as observed by an m/z shift of 42.01 for the sodiated singly acetylated and 42.01 for the protonated doubly acetylated. The mass increase of the protonated singly acetylated species was 42.00 (Figure 7). Control reactions containing all of the reaction components except AlgX, did not result in acetylated alginate even though 4-nitrophenyl acetate exhibited low spontaneous hydrolysis at pH 7.0 resulting in the production of 4-nitrophenyl and acetate. Neutral, commercially available sugars, cellohexose, xylohexose and maltotriose were not O-acetylated in the presence of AlgX. While both AlgJ and AlgX are necessary for alginate O-acetylation *in vivo* [13], [33] our binding and acetyltransferase data suggest AlgJ and AlgX do not have overlapping functions. Our current *in vitro* data provide additional evidence to support previous *in vivo* studies that there is no redundancy in the alginate acetylation machinery [31]–[33].

## Discussion

The O-acetylation of alginate requires the concerted action of four proteins: the putative MBOAT protein, AlgI; a protein of unknown function, AlgF; and two annotated O-acetyltransferases AlgJ and AlgX. To shed light on the role of each O-acetyltransferase, we determined the structure of *Pp*AlgJ_75–370_ and compared it with the recently solved structure of *Pa*AlgX_27–474_.Additionally, we kinetically characterized the acetylesterase activity of AlgJ and tested the ability of AlgJ and AlgX to bind and O-acetylate short polymannuronic acid oligomers.

The structure of *Pp*AlgJ_75–370_ reveals a fold that is structurally comparable to AlgX_44–344_ and other SGNH hydrolases. *Pp*AlgJ, like AlgX, is best described as an SGNH hydrolase-like protein as they both exhibit several key differences to canonical SGNH hydrolases. Not only is the order of the catalytic residues circularly permuted, but both proteins contain a cap domain and two long anti-parallel β-strands on one side of the protein that are not observed in other SGNH members. Although the function of the two long anti-parallel β-strands is currently unknown, given the involvement of other proteins in the O-acetylation system it is tempting to speculate that these regions may be involved in protein-protein interactions. A prediction for potential interaction surfaces on AlgJ was made using the consensus Protein-Protein Interaction Site Predictor (cons-PPISP) [46], [47]. Two predicted clusters of residues had positive scores for a possible interface. One cluster is comprised of residues 353–363 and is located on a loop and the beginning of α10 on the C-terminal end of the protein. A second cluster comprised of residues P79, G80, V81, D235 and F239 in *Pp*AlgJ is localized to the cap domain, located above the SGNH core. This cluster contains the conserved AlgJ signature motif residues that are imperative for alginate O-acetylation. The structure of *Pp*AlgJ has allowed us to confidently define the location of these residues, and propose roles for their function in O-acetylation. Signature residue variants in *Pa*AlgJ; P135A, K137A, D193A, H195A and W196F (*Pp*AlgJ equivalents; P132, K134, D190, H192 and W193) ablate O-acetylation [33]. The structure of *Pp*AlgJ, in addition to our kinetic data, indicates that D190 and H192 are part of the catalytic triad. Therefore, alanine variants D190A and H192A would disrupt the proposed catalytic serine by increasing the p*K*_a_ of the nucleophile and altering its orientation in the active site. The relatively rare internal lysine, K134 uses hydrogen bonding to stabilize the backbone oxygens of L187, R188 and D190 that form a loop between helices α_3_ and α_4_. It is expected that the loss of hydrogen bonding in the K134A variant disrupts the proper positioning of D190 in the DxH motif required for catalysis. The P132A variant located proximal to K134 would alter the secondary structure of, and impede proper function of K134. Lastly, the W193A variant is anticipated to disrupt the hydrophobic interactions with its neighbouring residues. Given that W193 is located proximal to catalytic H192, structural perturbations caused by disruption to the hydrophobic interactions are expected to have a negative impact on the catalytic triad.

Our *in vitro* enzymatic analysis probed the ability of *Pa*AlgJ and *Pp*AlgJ to perform the initial acetylesterase step of the overall acetyltransferase reaction. The results indicate that both AlgJ enzymes exhibit comparable catalytic parameters to AlgX for the hydrolysis of acetate (acetylesterase) from 3-carboxyumbelliferyl acetate [32]. Catalytic variants reduced acetylesterase activity by >80%. Although, the complete loss of activity was observed when similar catalytic residues were replaced in AlgX, *in vitro* residual activity in catalytic variants has been reported in at least one SGNH esterase [40]. The precise reason for residual activity is not clear since there are limited *in vitro* studies characterizing catalytic triad variants in SGNH hydrolase superfamily members. However, one possibility is the surrogate acetyl-donor tested does not optimally mimic the native acetyl donor of AlgJ. Residual hydrolysis of esterase substrates may also be attributed to surface amino acid residues that form microenvironments that promote spontaneous, non-enzymatic hydrolysis. For example, non-enzymatic hydrolysis has been reported for the SGNH superfamily member glutathione-S-transferase [48] and in the non-enzymatic protein albumin [49], [50]. The observation that the catalytic triad variants ablate O-acetylation *in vivo* further supports the notion that non-specific hydrolysis occurs *in vitro*.

**Figure 8:**
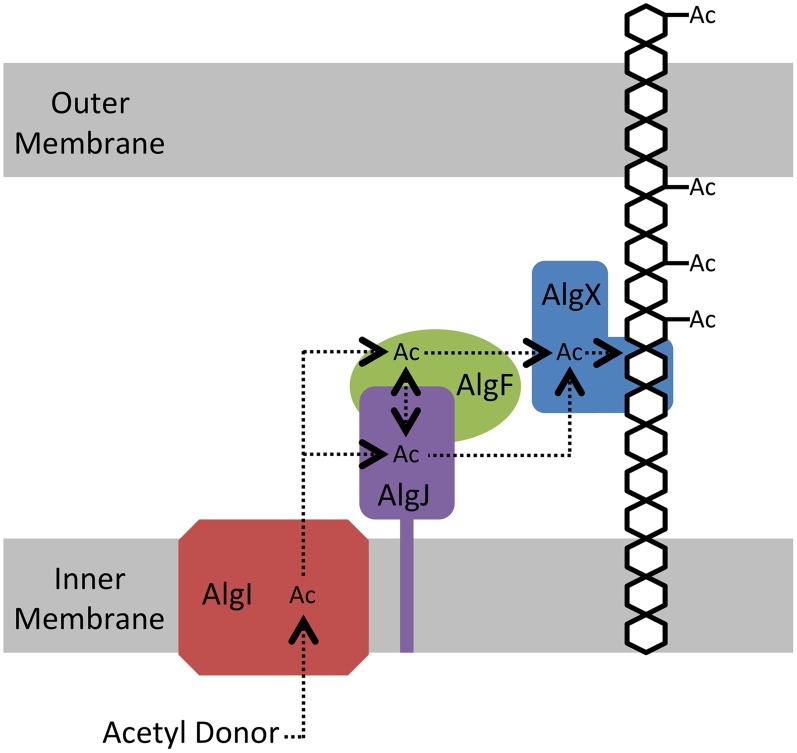
Proposed model for the *O*-acetylation of alginate. AlgI receives the currently unidentified acetyl donor molecule on its cytoplasmic face and shuttles the acetate or acetyl donor molecule through the inner membrane, where it is transferred to either AlgJ and/or AlgF. The acetyl donor may or may not be transferred between AlgJ and AlgF. From its intermediate location, the acetyl donor molecule is transferred to AlgX, which subsequently *O*-acetylates the alginate polymer. Although absolutely required for alginate *O*-acetylation the function of AlgF is currently unknown. We have demonstrated that AlgJ has activity on acetylated substrates thereby suggesting it fulfills a more direct role in the O-acetylation process.

The electrostatic surfaces of *Pp*AlgJ_75–370_ and the SGNH hydrolase-like domain of *Pa*AlgX_27–474_ are distinctly different with respect to the active site region. *Pa*AlgX contains an electropositive groove that stretches from the active site to the C-terminal CBM [32]. We previously proposed that this highly conserved electropositive region would be compatible for binding the anionic alginate polymer. A direct ESI-MS binding assay confirmed that AlgX is able to bind mannuronic acid oligomers, with longer, more physiologically relevant polymers of mannuronic acid exhibiting higher affinity for the protein. The addition of a surrogate acetyl-donor in the presence of mannuronic acid oligomers led to the first demonstration *in vitro* that AlgX is an O-acetyltransferase capable of transferring acetyl groups to mannuronic acid oligomers. While ESI-MS cannot explicitly identify the location of carbohydrate-protein interaction, the ability for AlgX to O-acetylate mannuronic acid oligomers demonstrates that these oligosaccharides must, in part, bind to the active site. In comparison, *Pp*AlgJ demonstrated very weak or no affinity toward mannuronic acid oligosaccharides under the conditions tested. Taken together, these data suggest that AlgX may be the only enzyme that O-acetylates alginate and that it functions in a non-redundant, successive mechanism with the other proteins in the acetylation complex machinery. Our data allow us to propose an updated model for alginate O-acetylation (Figure 8). Briefly, AlgI interacts with an unknown acetyl donor molecule in the cytoplasm and transfers acetate or acetyl donor across the inner membrane to either AlgJ and/or AlgF in the periplasm. The transmembrane domain of AlgJ (residues 12–29) tethers the enzyme to the membrane and potentially closer to AlgI than either AlgF or AlgX which lack this spatial constraint. Given the current state of knowledge, the function and mechanism of the intermediate step(s) involving AlgJ or AlgF can only be speculated. The order of transfer of the acetyl donor is uncertain, but the fact that AlgJ can perform the initial esterase stage of transferase reaction suggests that, given the right donor, recipient, and environment, this enzyme is directly involved in the O-acetylation process. This possibility cannot be ruled out for AlgF either, but the lack of identifiable catalytic residues in this protein suggests that it may serve a more accessory role. However, it is clear that AlgX can catalyze the direct O-acetylation of alginate. Thus, it is conceivable that AlgX receives the acetate or acetyl donor molecule from either AlgJ or AlgF, and then subsequently catalyzes the transfer of this substrate to O-acetylate alginate. This model is supported by the observation that active site variants of either AlgJ or AlgX are sufficient to abolish acetylation *in vivo* [32], [33]. In addition, the data presented herein exclude the possibility that both enzymes can O-acetylate alginate.

Although the O-acetylation of polysaccharides requires a minimum of two functionalities, O-acetylation of alginate and cellulose requires four distinct proteins including a membrane associated and non-membrane associated periplasmic O-acetyltransferase. In contrast, the O-acetylation of peptidoglycan utilizes a single O-acetyltransferase that may not be constrained to the inner membrane depending on the presence of an N-terminal transmembrane domain. Regardless of the number of proteins involved in the system, the catalytic mechanism of acetate transfers across the inner membrane and the acetylation of the polymer is undoubtedly highly conserved. Additionally, the role of O-acetylation of these polysaccharides in both Gram-negative and Gram-positive bacteria is functionally similar, allowing for protection against external agents [16]–[21], [26], [27]. Intuitively, the requirement of four proteins involved in relaying acetate from the cytosol to the polysaccharide appears inefficient when compared to the O-acetylation of peptidoglycan. While additional proteins could facilitate increased regulation, the extent of O-acetylation of peptidoglycan and alginate between species, strain, and culture conditions, varies between 20–70% (relative to muramic acid content) and 4–57%, respectively [51], [52]. This indicates that the amount of O-acetylation cannot be simply categorized based on the number of proteins involved or their localization.

A defining characteristic of cellulose and alginate biosynthesis compared to peptidoglycan is that these polysaccharides must traverse two membranes for export before reaching their final location, which in turn requires the involvement of several additional proteins in polymer modification and export. Therefore, four O-acetylation proteins may be an inherent requirement to adapt the O-acetylation machinery to the biosynthetic export machinery. In support of this, AlgX has been demonstrated to interact with both outer membrane alginate export proteins and periplasmic proteins required in alginate modification [53]–[57]. Such interactions have not been observed in the O-acetylation of peptidoglycan. The proteins WssFGHI are compulsory for cellulose acetylation [58] and WssGHI are homologous to AlgFIJ, with amino acid sequence identities of 24, 46, and 33%, respectively. We have previously suggested that WssF, which is predicted to belong to the SGNH hydrolase superfamily may be analogous to AlgX although it lacks the CBM present in AlgX [32]. Since proteins involved in cellulose acetylation have not been studied at either the structural or functional level, our present studies on AlgJ and AlgX are significant as they provide further data to support the roles of these proteins beyond sequence homology and phenotypic analysis.

We have successfully determined the structure of AlgJ, a protein involved in the alginate *O*-acetylation pathway. AlgJ exhibits acetylesterase activity that is mediated through the Ser-His-Asp catalytic triad similar to that of the SGNH hydrolase-like enzyme AlgX. ESI-MS confirmed that AlgX but not AlgJ binds polymannuronic acid and we have conclusively demonstrated that AlgX is an O-acetyltransferase, and that it is the only annotated O-acetyltransferase in the pathway that can both interact with, and O-acetylate alginate. Refining the model for alginate O-acetylation provides new avenues for further studies into the mechanism of O-acetylation of polysaccharides in the microbial kingdom.

## Materials and Methods

### Chemicals, bacterial strains, plasmids, and growth media

Superflow Ni^2+^ NTA-agarose resin was obtained from Qiagen (Mississauga, ON). Graphitized carbon solid phase extraction columns (Carbograph SPE) are products of Grace Canada, Inc (Ajax, ON). All other chemicals and reagents, unless otherwise stated, were supplied by Sigma-Aldrich Canada Ltd. (Oakville, ON). All growth media was obtained from Bio Basic (Markham, ON). DNA manipulations were performed in *E. coli* DH5α and protein expression of the SeMet protein was carried out using *E. coli* B834 (DE3) Met-auxotroph cells and grown in media supplemented with kanamycin at 50 µg mL^−1^. Protein expression for binding and enzyme assays was carried out in *E. coli* BL21-CodonPlus cells.

### DNA manipulations

The nucleotide sequences of *algJ* from *P. putida* KT2440 and *P. aeruginosa* PAO1 were acquired from the *Pseudomonas* Genome Database [59]. The boundaries of the putative *O*-acetyltransferase domain of *P. aeruginosa* AlgJ were predicted to range from amino acids 79 to 379 (*Pa*AlgJ_79–379_) based on Phyre^2^ [60] structural alignments using the N-terminal domain of *P. aeruginosa* AlgX (*Pa*AlgX_44–344_) [32]. Sequence alignment revealed that these boundaries correspond to amino acids 75 to 370 in the *P. putida* AlgJ homolog (*Pp*AlgJ_75–370_). PCR amplification was carried out using the high fidelity DNA polymerase, PfuTurbo (Stratagene). FastDigest restriction enzymes were obtained from Fermentas. Plasmid DNA was extracted from *E. coli* using the PureLink Quick plasmid miniprep kit from Invitrogen (Burlington, ON). Primer sequences used in the generation of wild type (WT) *Pa*AlgJ_79–379_ and *Pa*AlgX_44–344_, as well as proposed active site mutants are summarized in Supplementary Table S1. The PCR reaction conditions were as follows: *Pfu* buffer with 2 mM MgSO_4_ (Thermo Scientific), 10 ng template DNA, 10 ng forward and reverse primer each, 25 µM dNTPs, 2 mM MgSO_4_, 2.5 U *Pfu* polymerase (Thermo Scientific) in a total reaction volume of 25 µL. The PCR product was digested with *Nde*I and *Xho*I and ligated into a pET28a vector backbone to generate (i) a thrombin cleavable N-terminal hexahistidine tag (His_6_) construct; and (ii) a second construct used for crystallography that contained both an N and C-terminal His_6_-tag. Site directed mutagenesis was performed using the QuikChange Lightning kit according to the prescribed protocol (Agilent Technologies). Constructs generated were verified by sequencing performed by ACGT DNA Technologies Corporation (Toronto, ON).

### Expression and purification of AlgJ

The expression and purification of native wild type and mutant *Pa*AlgJ_79–379_ and *Pp*AlgJ_75–370_ constructs were identical. SeMet *Pp*AlgJ_75–370_ was used for structure determination and was expressed using the protocol described by Lee *et al* [61]. The expression and purification protocol was as follows: Starter cultures were grown overnight in 50 mL Luria-Bertani (LB) broth containing 50 µg mL^−1^ kanamycin at 310 K in a shaking incubator, with *E. coli* BL21-CodonPlus cells transformed with the appropriate plasmid. The cells were subsequently inoculated into 1 L LB broth containing 50 µg mL^−1^ kanamycin at 310 K in a shaking incubator. Upon reaching an OD_600_ of 0.7, the cells were induced with isopropyl β-D-1-thiogalactopyranoside (IPTG) to a final concentration of 1 mM. The induced cells were allowed to grow for an additional 18 h at 291 K. The cells were harvested via centrifugation at 5000× *g* for 20 min at 277 K. The cell pellet was stored at 253 K until needed. Frozen cell pellet was thawed over ice and re-suspended in 50 mL cold lysis buffer (500 mM NaCl, 20 mM Tris-HCl pH 8.0) containing one SIGMAFAST EDTA-free protease-inhibitor cocktail tablet (Sigma). The cells were homogenized at 10,000 psi through an Emulsiflex C3 (Avestin Inc.) with at least 3 passes until uniform in consistency. The resultant cell lysate was centrifuged at 25000× *g* for 25 minutes at 278 K to pellet cell debris and insoluble material. The soluble cell lysate was loaded onto a 5 mL Ni^2+^-NTA gravity column equilibrated with Ni-NTA buffer (500 mM NaCl, 20 mM Tris-HCl pH 8.0, 5 mM imidazole). The column was washed with 10 column volumes of Ni-NTA buffer containing 30 mM imidazole. Protein bound to the column was eluted with 4 column volumes of Ni-NTA buffer containing 150 mM imidazole. The eluent was dialyzed against 4 L of S200 buffer (150 mM NaCl, 20 mM Tris-HCl pH 8.0). Protein concentration was measured using the Pierce BCA Protein Assay Kit from Thermo Scientific (Rockford, IL). The His_6_-tag was cleaved from the protein via incubation with thrombin (Novagen) at 0.5 U per mg of protein at 298 K for at least 2 h. The thrombin treated protein was loaded onto a 1 mL Ni^2+^-NTA column equilibrated with S200 buffer containing 5 mM imidazole. The column was washed with 10 column volumes of S200 buffer containing 30 mM imidazole. The initial flow through and wash were pooled together and contained the un-tagged protein. The untagged protein was concentrated to a 1–2 mL volume using an Amicon Ultra centrifugation filter device (Milipore) with a 30 kDa cutoff. Approximately 20 mg of purified protein could be obtained per litre of bacterial culture. The concentrated protein was further purified via size exclusion chromatography on a HiLoad 16/60 Superdex 200 gel filtration column (GE Healthcare). Fractions containing protein were pooled and analyzed via SDS-PAGE to be >95% pure. *Pa*AlgJ_79–379_ and *Pp*AlgJ_75–370_ protein could be stored at 277 K for up to 2 or 4 weeks, respectively, before significant degradation was observed by SDS-PAGE.

### Crystallization and structure determination of *Pp*AlgJ_75–370_

SeMet *Pp*AlgJ_75–370_ was concentrated to ∼6–8 mg mL^−1^ by an Amicon Ultrafiltration device (30 kDa MWCO, Milipore) for crystallization trials. Sparse-matrix screens were setup by hand using MCSG suites 1–4 (Microlytic) in 48-well VDX plates (Hampton Research). The drops consisted of a 1∶1 ratio of protein to well solution at a final volume of 4 µL equilibrated over 250 µL of well solution, and stored at 293 K. Numerous hits were obtained after 1 week and were primarily found in conditions containing a divalent cation (Mg^2+^ or Ca^2+^) and polyethylene glycol (PEG) solutions between 3350–6000 Da. In most cases the crystals nucleated from a single point and radiated outwards forming a cluster with individual crystals estimated to be a maximum of 500 µm, and were of diffraction quality directly from the sparse matrix screens.

Crystals used for data collection were found in MCSG-1, condition 7 (0.2 M MgCl_2_, 0.1 M Bis-Tris∶HCl pH 5.5, 25% (w/v) PEG3350). Prior to data collection, crystals were cryo-protected by exchanging the drop solution with cryo-protectant solution (0.2 M MgCl_2_, 0.1 M Bis-Tris∶HCl pH 5.5, 25% (w/v) PEG3350, 20% (v/v) ethylene glycol). The exchange was performed through the addition of cryo-protectant solution directly to the drop and the removal of the added volume until complete exchange had occurred. The crystal clusters were disrupted via physical contact with a fine needle until an isolated single crystal could be looped. Crystals were vitrified in liquid nitrogen and stored. Selenium single-wavelength anomalous dispersion (Se-SAD) X-ray diffraction data were collected on beamline X29A at the National Synchrotron Light Source (NSLS) at Brookhaven National Laboratory. 90 images at 94% beam attenuation with 2° Δφ oscillation and 360 images without beam attenuation with 1° Δφ oscillation were collected on an ADSC Q315 CCD detector at a 260 mm crystal-to-detector distance and 0.4 s exposure time per image. The Se-SAD data was indexed, integrated, scaled and merged using HKL-2000 (Table 1) [62], and used in conjunction with HKL2MAP to locate 12 (of 14) selenium sites, with density modified phases calculated using SOLVE/RESOLVE [63]. The electron density maps were of sufficient quality for automatic model building using PHENIX AutoBuild [64] and subsequent manual model building using COOT [65], [66]. Model refinement was performed using PHENIX.REFINE [67] and progress was monitored as a function of the reduction and convergence of *R*_work_ and *R*_free_ (Table 1) [66]. TLS groups were added to the refinement in PHENIX through the use of the TLSMD server [68].

### Structural and bioinformatics analysis of AlgJ

All figures that display the structure of *Pp*AlgJ_75–370_ and/or *Pa*AlgX_27–474_ were generated using PyMol (The PyMol Molecular Graphics System, version 1.6.0.0, Schrödinger, LLC). The secondary structure was determined using the STRIDE web server [69]. Surface representations demonstrating either electrostatics or surface residue conservation were depicted with all side chains present even if they could not be accurately modeled in the structure. Electrostatic surfaces were generated using the ABPS Tools 2.1 plugin that is integrated into PyMol [70]. Surface residue conservation was determined using the ConSurf web server [71] and visualized using the provided color scheme. Solvent accessible surface area was calculated using the PDBePISA server [72]. Surface residue conservation was determined using the ConSurf web server [71] and visualized using the provided color scheme. Solvent accessible surface area was calculated using the PDBePISA server [72].

Sequence alignments to determine AlgJ homologs were determined using BLAST [73]. Multiple sequence alignments were generated using Multiple Sequence Comparison by Log-Expectation (MUSCLE) [74], [75]. Prediction of potential protein-protein interaction surfaces was performed using cons-PPISP [46], [47].

### Enzyme assay

All enzyme assays were performed at least in triplicate, in a 96-well microtiter plate, using a SpectraMax M2 from Molecular Devices (Sunnyvale, CA). Standard reactions contained 3.0 mM 3-carboxyumbelliferyl acetate (ACC), dissolved in DMSO for specific activity assays and variable concentrations ranging between 0.1 *K*_m_ and ≥2 *K*_m_ and 30 µg each wild-type protein and 100 µg for each variant in a total volume of 100 µL in 50 mM sodium HEPES buffer (pH 7.0 for AlgJ, pH 8.0 for AlgX) at 298 K. The final DMSO concentration did not exceed 10% (v/v). Due to low substrate solubility, reactions with higher substrate concentrations could not be obtained. Reactions were initiated by the addition of substrate and reactions were monitored in real time for a duration of 10 min using an excitation of 386 nm and an emission of 447 nm as previously described [32]. The hydrolysis and release of acetate results in an increase in the fluorescence signal. Background hydrolysis rates, in the absence of enzyme, were monitored and subtracted from enzyme-catalyzed reactions. A calibration curve for 7-hydroxycourmarin-3-carboxylic acid, the fluorescent hydrolysis product of 3-carboxyumbelliferyl acetate, was obtained under the reaction conditions and used to calculate reaction rate. The protein concentration of each enzyme variant was determined using the Pierce BCA Protein Assay Kit from Thermo Scientific (Rockford, IL). Data were fit by nonlinear regression to the Michealis-Menten equation using GraphPad Prism 6.0c for Mac, (GraphPad Software, La Jolla California USA, www.graphpad.com).

### Alginate binding assay

Immediately prior to ESI-MS analysis, *Pa*AlgX_27–474_ and *Pa*AlgJ_79–379_ were each dialyzed against aqueous 100 mM ammonium acetate (pH 7.0) using microconcentrators (Millipore Corp., Bedford, MA) with a MW cut-off of 30 kDa (for AlgX) and 10 kDa (for AlgJ). Two different reference proteins (P_ref_) were used to correct ESI mass spectra for the occurrence of nonspecific carbohydrate-protein binding (during the ESI process): a single chain fragment (scFv, MW 26 539 Da) of the monoclonal antibody (mAb) Se155-4, which was produced and purified as described previously [76], and lysozyme (Lyz, MW 14 300 Da), which was obtained from Sigma-Aldrich Canada (Oakville, Canada) and used without further purification. Each protein was concentrated and dialyzed against 50 mM ammonium acetate using microconcentrators (Millipore Corp., Bedford, MA) with a MW cut-off of 10 kDa and stored at −20°C until needed. Stock solutions of each of the polymannurnic acid oligomers, tetramer through dodecamer (ManA_4_ – ManA_12_), and undeca- and pentadeca-hyaluronic acid saccharides (HA_11_ and HA_15_), synthesized as previously described [77], [78] were prepared by dissolving known amounts of solid compound in ultrafiltered water (Milli-Q, Millipore, Bedford, MA) to give a final concentration of ∼1 mM. The ligand solutions were stored at 253 K until needed.

Affinity measurements were carried out on a Synapt G2 quadrupole-ion mobility separation-time of flight (Q-IMS-TOF) mass spectrometer (Waters, UK) equipped with a modified nanoflow ESI (nanoESI) source. Complete details of the instrumental and experimental conditions used for the ESI-MS binding measurements along with descriptions of how the data were analyzed to establish association constants (*K*_a_) for protein-ligand interactions have been described previously [79]–[81].

### O-Acetyltransferase assay

*O*-Acetyltransferase assays were completed in a similar fashion as those previously described for the O-acetylation of peptidoglycan with modifications [51]. Briefly, 3 mM 4-nitrophenyl acetate dissolved in ethanol (3% final concentration), and 1 mM of ManA_10_ [78], was incubated with 60 µg of AlgX in 50 mM sodium phosphate buffer, pH 7.0 for a duration of 1 h at 298 K. Control reactions containing 1 mM of cellohexose, xylohexose and maltotriose in place of alginate were also completed. All reactions were repeated in the absence of AlgX as a negative control. The total reaction volume was 150 µL and the reaction was initiated by the addition of protein. The reactions were quenched by applying the entire reaction to a 4 mL graphitized carbon solid phase extraction column previously washed with three column volumes of 100% acetonitrile containing 0.1% (v/v) trifluoroacetic acid (TFA) and then equilibrated with three column volumes of water. Following application of the reaction, the column was washed successively with three column volumes of 100% acetonitrile, 100% acetonitrile containing 0.1% (v/v) TFA, 3∶1 isopropyl alcohol: acetonitrile (v/v). Polymannuronic acids were eluted with 6 mL of 50% tetrohydrofuran containing 0.1% TFA (v/v) and dried under vacuum in a Speed Vac at 298 K. The dried samples were resuspended in 100 µL of water and stored at 253 K prior to analysis.

Liquid chromatography–mass spectrometry analyses were performed on an Agilent 1200 HPLC liquid chromatograph interfaced with an Agilent UHD 6530 Q-Tof mass spectrometer at the Mass Spectrometry Facility of the Advanced Analysis Centre, University of Guelph. A C18 column (Agilent Poroshell 120, 150 mm×4.6 mm 2.7 µm) was used for chromatographic separation with solution A (0.1% formic acid) and solution B (100% acetonitrile with 0.1% formic acid). The mobile phase gradient was as follows: initial conditions, 2% solution B increasing to 98% solution B in 30 min followed by a column wash at 98% solution B and 10 minute re-equilibration. The first 2 and last 5 minutes of gradient were sent to waste. The flow rate was maintained at 0.1 mL/min. The mass spectrometer electrospray capillary voltage was maintained at 4.0 kV and the drying gas temperature at 523 K with a flow rate of 8 L/min. Nebulizer pressure was 30 psi and the fragmentor was set to 160. Nitrogen was used as both the nebulizing and drying gas, and the collision-induced gas. The mass-to-charge ratio was scanned across the m/z range of 100–3000 m/z in 4 GHz (extended dynamic range positive-ion auto MS/MS mode). Three precursor ions per cycle were selected for fragmentation. The instrument was externally calibrated with the ESI TuneMix (Agilent). The sample injection volume was 10 µl. The resultant spectra were analyzed using mMass software (http://www.mmass.org/).

### Accession numbers

The coordinates and structure factors for *Pp*AlgJ_75–370_ have been deposited in the PDB, ID code 4O8V.

## Supporting Information

Table S1 Bacterial strains and plasmids used in this study.
(DOCX)

## References

[1] Leid JG, Willson CJ, Shirtliff ME, Hassett DJ, Parsek MR, et al (2005) The exopolysaccharide alginate protects Pseudomonas aeruginosa biofilm bacteria from IFN-gamma-mediated macrophage killing. J Immunol 175: 7512–7518. 16301659 10.4049/jimmunol.175.11.7512

[2] Davies D (2003) Understanding biofilm resistance to antibacterial agents. Nat Rev Drug Discov 2: 114–122. 12563302 10.1038/nrd1008

[3] Konig B, Friedl P, Pedersen SS, Konig W (1992) Alginate–its role in neutrophil responses and signal transduction towards mucoid Pseudomonas aeruginosa bacteria. Int Arch Allergy Immunol 99: 98–106. 1336422 10.1159/000236341

[4] Grobe KJ, Zahller J, Stewart PS (2002) Role of dose concentration in biocide efficacy against Pseudomonas aeruginosa biofilms. J Ind Microbiol Biotechnol 29: 10–15. 12080421 10.1038/sj.jim.7000256

[5] Nichols WW, Dorrington SM, Slack MP, Walmsley HL (1988) Inhibition of tobramycin diffusion by binding to alginate. Antimicrob Agents Chemother 32: 518–523. 3132093 10.1128/aac.32.4.518 PMC172213

[6] Colvin KM, Gordon VD, Murakami K, Borlee BR, Wozniak DJ, et al (2011) The pel polysaccharide can serve a structural and protective role in the biofilm matrix of Pseudomonas aeruginosa. PLoS Pathog 7: e1001264. 21298031 10.1371/journal.ppat.1001264 PMC3029257

[7] Khan W, Bernier SP, Kuchma SL, Hammond JH, Hasan F, et al (2010) Aminoglycoside resistance of Pseudomonas aeruginosa biofilms modulated by extracellular polysaccharide. Int Microbiol 13: 207–212. 21404215 10.2436/20.1501.01.127 PMC3721063

[8] Franklin MJ, Nivens DE, Weadge JT, Howell PL (2011) Biosynthesis of the Pseudomonas aeruginosa Extracellular Polysaccharides, Alginate, Pel, and Psl. Front Microbiol 2: 167. 21991261 10.3389/fmicb.2011.00167 PMC3159412

[9] Colvin KM, Irie Y, Tart CS, Urbano R, Whitney JC, et al (2012) The Pel and Psl polysaccharides provide Pseudomonas aeruginosa structural redundancy within the biofilm matrix. Environ Microbiol 14: 1913–1928. 22176658 10.1111/j.1462-2920.2011.02657.x PMC3840794

[10] May TB, Shinabarger D, Maharaj R, Kato J, Chu L, et al (1991) Alginate synthesis by Pseudomonas aeruginosa: a key pathogenic factor in chronic pulmonary infections of cystic fibrosis patients. Clin Microbiol Rev 4: 191–206. 1906371 10.1128/cmr.4.2.191 PMC358191

[11] Ohman DE, Chakrabarty AM (1982) Utilization of human respiratory secretions by mucoid Pseudomonas aeruginosa of cystic fibrosis origin. Infect Immun 37: 662–669. 6811437 10.1128/iai.37.2.662-669.1982 PMC347583

[12] Franklin MJ, Ohman DE (1993) Identification of algF in the alginate biosynthetic gene cluster of Pseudomonas aeruginosa which is required for alginate acetylation. J Bacteriol 175: 5057–5065. 8394313 10.1128/jb.175.16.5057-5065.1993 PMC204972

[13] Franklin MJ, Ohman DE (2002) Mutant analysis and cellular localization of the AlgI, AlgJ, and AlgF proteins required for O acetylation of alginate in Pseudomonas aeruginosa. J Bacteriol 184: 3000–3007. 12003941 10.1128/JB.184.11.3000-3007.2002 PMC135050

[14] Gacesa P (1998) Bacterial alginate biosynthesis–recent progress and future prospects. Microbiology 144 (Pt 5) 1133–1143. 9611788 10.1099/00221287-144-5-1133

[15] Linker A, Jones RS (1964) A Polysaccharide Resembling Alginic Acid from a Pseudomonas Micro-Organism. Nature 204: 187–188. 14222269 10.1038/204187a0

[16] Vuong C, Kocianova S, Voyich JM, Yao Y, Fischer ER, et al (2004) A crucial role for exopolysaccharide modification in bacterial biofilm formation, immune evasion, and virulence. J Biol Chem 279: 54881–54886. 15501828 10.1074/jbc.M411374200

[17] Cerca N, Jefferson KK, Maira-Litran T, Pier DB, Kelly-Quintos C, et al (2007) Molecular basis for preferential protective efficacy of antibodies directed to the poorly acetylated form of staphylococcal poly-N-acetyl-beta-(1–6)-glucosamine. Infect Immun 75: 3406–3413. 17470540 10.1128/IAI.00078-07 PMC1932961

[18] Itoh Y, Rice JD, Goller C, Pannuri A, Taylor J, et al (2008) Roles of pgaABCD genes in synthesis, modification, and export of the Escherichia coli biofilm adhesin poly-beta-1,6-N-acetyl-D-glucosamine. J Bacteriol 190: 3670–3680. 18359807 10.1128/JB.01920-07 PMC2394981

[19] Wang X, Preston JF 3rd, Romeo T (2004) The pgaABCD locus of Escherichia coli promotes the synthesis of a polysaccharide adhesin required for biofilm formation. J Bacteriol 186: 2724–2734. 15090514 10.1128/JB.186.9.2724-2734.2004 PMC387819

[20] Colvin KM, Alnabelseya N, Baker P, Whitney JC, Howell PL, et al (2013) PelA deacetylase activity is required for Pel polysaccharide synthesis in Pseudomonas aeruginosa. J Bacteriol 195: 2329–2339. 23504011 10.1128/JB.02150-12 PMC3650530

[21] Wan Z, Brown PJ, Elliott EN, Brun YV (2013) The adhesive and cohesive properties of a bacterial polysaccharide adhesin are modulated by a deacetylase. Mol Microbiol 88: 486–500. 23517529 10.1111/mmi.12199 PMC3633684

[22] Gille S, Pauly M (2012) O-acetylation of plant cell wall polysaccharides. Front Plant Sci 3: 12. 22639638 10.3389/fpls.2012.00012 PMC3355586

[23] Moynihan PJ, Sychantha D, Clarke AJ (2014) Chemical biology of peptidoglycan acetylation and deacetylation. Bioorg Chem 54C: 44–50. 10.1016/j.bioorg.2014.03.010 24769153

[24] Vollmer W (2008) Structural variation in the glycan strands of bacterial peptidoglycan. FEMS Microbiol Rev 32: 287–306. 18070068 10.1111/j.1574-6976.2007.00088.x

[25] Weadge JT, Clarke AJ (2007) Neisseria gonorrheae O-acetylpeptidoglycan esterase, a serine esterase with a Ser-His-Asp catalytic triad. Biochemistry 46: 4932–4941. 17388571 10.1021/bi700254m

[26] Moynihan PJ, Clarke AJ (2011) O-Acetylated peptidoglycan: controlling the activity of bacterial autolysins and lytic enzymes of innate immune systems. Int J Biochem Cell Biol 43: 1655–1659. 21889603 10.1016/j.biocel.2011.08.007

[27] Moynihan PJ, Clarke AJ (2010) O-acetylation of peptidoglycan in gram-negative bacteria: identification and characterization of peptidoglycan O-acetyltransferase in Neisseria gonorrhoeae. J Biol Chem 285: 13264–13273. 20178982 10.1074/jbc.M110.107086 PMC2857125

[28] Bernard E, Rolain T, Courtin P, Guillot A, Langella P, et al (2011) Characterization of O-acetylation of N-acetylglucosamine: a novel structural variation of bacterial peptidoglycan. J Biol Chem 286: 23950–23958. 21586574 10.1074/jbc.M111.241414 PMC3129176

[29] Bera A, Herbert S, Jakob A, Vollmer W, Gotz F (2005) Why are pathogenic staphylococci so lysozyme resistant? The peptidoglycan O-acetyltransferase OatA is the major determinant for lysozyme resistance of Staphylococcus aureus. Mol Microbiol 55: 778–787. 15661003 10.1111/j.1365-2958.2004.04446.x

[30] Pier GB, Coleman F, Grout M, Franklin M, Ohman DE (2001) Role of alginate O acetylation in resistance of mucoid Pseudomonas aeruginosa to opsonic phagocytosis. Infect Immun 69: 1895–1901. 11179370 10.1128/IAI.69.3.1895-1901.2001 PMC98099

[31] Franklin MJ, Ohman DE (1996) Identification of algI and algJ in the Pseudomonas aeruginosa alginate biosynthetic gene cluster which are required for alginate O acetylation. J Bacteriol 178: 2186–2195. 8636017 10.1128/jb.178.8.2186-2195.1996 PMC177924

[32] Riley LM, Weadge JT, Baker P, Robinson H, Codee JD, et al (2013) Structural and functional characterization of Pseudomonas aeruginosa AlgX: role of AlgX in alginate acetylation. J Biol Chem 288: 22299–22314. 23779107 10.1074/jbc.M113.484931 PMC3829321

[33] Franklin MJ, Douthit SA, McClure MA (2004) Evidence that the algI/algJ gene cassette, required for O acetylation of Pseudomonas aeruginosa alginate, evolved by lateral gene transfer. J Bacteriol 186: 4759–4773. 15231808 10.1128/JB.186.14.4759-4773.2004 PMC438637

[34] Riley LM, Weadge JT, Baker P, Robinson H, Codee JD, et al (2013) Structural and functional characterization of Pseudomonas aeruginosa AlgX: role of AlgX in alginate acetylation. J Biol Chem 288: 22299–22314. 23779107 10.1074/jbc.M113.484931 PMC3829321

[35] Nivens DE, Ohman DE, Williams J, Franklin MJ (2001) Role of alginate and its O acetylation in formation of Pseudomonas aeruginosa microcolonies and biofilms. J Bacteriol 183: 1047–1057. 11208804 10.1128/JB.183.3.1047-1057.2001 PMC94973

[36] Krogh A, Larsson B, von Heijne G, Sonnhammer EL (2001) Predicting transmembrane protein topology with a hidden Markov model: application to complete genomes. J Mol Biol 305: 567–580. 11152613 10.1006/jmbi.2000.4315

[37] Akoh CC, Lee GC, Liaw YC, Huang TH, Shaw JF (2004) GDSL family of serine esterases/lipases. Prog Lipid Res 43: 534–552. 15522763 10.1016/j.plipres.2004.09.002

[38] Lescic Asler I, Ivic N, Kovacic F, Schell S, Knorr J, et al (2010) Probing enzyme promiscuity of SGNH hydrolases. Chembiochem 11: 2158–2167. 20931591 10.1002/cbic.201000398

[39] Hedstrom L (2002) Serine protease mechanism and specificity. Chem Rev 102: 4501–4524. 12475199 10.1021/cr000033x

[40] Pfeffer JM, Weadge JT, Clarke AJ (2013) Mechanism of action of Neisseria gonorrhoeae O-acetylpeptidoglycan esterase, an SGNH serine esterase. J Biol Chem 288: 2605–2613. 23209280 10.1074/jbc.M112.436352 PMC3554927

[41] Konermann L, Douglas DJ (1998) Unfolding of proteins monitored by electrospray ionization mass spectrometry: a comparison of positive and negative ion modes. J Am Soc Mass Spectrom 9: 1248–1254. 9835071 10.1016/S1044-0305(98)00103-2

[42] Grandori R (2003) Origin of the conformation dependence of protein charge-state distributions in electrospray ionization mass spectrometry. J Mass Spectrom 38: 11–15. 12526001 10.1002/jms.390

[43] Hamdy OM, Julian RR (2012) Reflections on charge state distributions, protein structure, and the mystical mechanism of electrospray ionization. J Am Soc Mass Spectrom 23: 1–6. 22076632 10.1007/s13361-011-0284-8

[44] Hall Z, Robinson CV (2012) Do charge state signatures guarantee protein conformations? J Am Soc Mass Spectrom 23: 1161–1168. 22562394 10.1007/s13361-012-0393-z

[45] Kaltashov IA, Abzalimov RR (2008) Do ionic charges in ESI MS provide useful information on macromolecular structure? J Am Soc Mass Spectrom 19: 1239–1246. 18602274 10.1016/j.jasms.2008.05.018

[46] Chen H, Zhou HX (2005) Prediction of interface residues in protein-protein complexes by a consensus neural network method: test against NMR data. Proteins 61: 21–35. 16080151 10.1002/prot.20514

[47] Zhou HX, Shan Y (2001) Prediction of protein interaction sites from sequence profile and residue neighbor list. Proteins 44: 336–343. 11455607 10.1002/prot.1099

[48] Satoh K (1995) The high non-enzymatic conjugation rates of some glutathione S-transferase (GST) substrates at high glutathione concentrations. Carcinogenesis 16: 869–874. 7728969 10.1093/carcin/16.4.869

[49] Ascenzi P, Gioia M, Fanali G, Coletta M, Fasano M (2012) Pseudo-enzymatic hydrolysis of 4-nitrophenyl acetate by human serum albumin: pH-dependence of rates of individual steps. Biochem Biophys Res Commun 424: 451–455. 22771811 10.1016/j.bbrc.2012.06.131

[50] Lockridge O, Xue W, Gaydess A, Grigoryan H, Ding SJ, et al (2008) Pseudo-esterase activity of human albumin: slow turnover on tyrosine 411 and stable acetylation of 82 residues including 59 lysines. J Biol Chem 283: 22582–22590. 18577514 10.1074/jbc.M802555200 PMC2504902

[51] Moynihan PJ, Clarke AJ (2013) Assay for peptidoglycan O-acetyltransferase: a potential new antibacterial target. Anal Biochem 439: 73–79. 23660013 10.1016/j.ab.2013.04.022

[52] Skjak-Braek G, Grasdalen H, Larsen B (1986) Monomer sequence and acetylation pattern in some bacterial alginates. Carbohydr Res 154: 239–250. 3098421 10.1016/s0008-6215(00)90036-3

[53] Hay ID, Schmidt O, Filitcheva J, Rehm BH (2012) Identification of a periplasmic AlgK-AlgX-MucD multiprotein complex in Pseudomonas aeruginosa involved in biosynthesis and regulation of alginate. Appl Microbiol Biotechnol 93: 215–227. 21713511 10.1007/s00253-011-3430-0

[54] Keiski CL, Harwich M, Jain S, Neculai AM, Yip P, et al (2010) AlgK is a TPR-containing protein and the periplasmic component of a novel exopolysaccharide secretin. Structure 18: 265–273. 20159471 10.1016/j.str.2009.11.015 PMC2857933

[55] Whitney JC, Hay ID, Li C, Eckford PD, Robinson H, et al (2011) Structural basis for alginate secretion across the bacterial outer membrane. Proc Natl Acad Sci U S A 108: 13083–13088. 21778407 10.1073/pnas.1104984108 PMC3156188

[56] Jain S, Ohman DE (2005) Role of an alginate lyase for alginate transport in mucoid Pseudomonas aeruginosa. Infect Immun 73: 6429–6436. 16177314 10.1128/IAI.73.10.6429-6436.2005 PMC1230907

[57] Jain S, Franklin MJ, Ertesvag H, Valla S, Ohman DE (2003) The dual roles of AlgG in C-5-epimerization and secretion of alginate polymers in Pseudomonas aeruginosa. Mol Microbiol 47: 1123–1133. 12581364 10.1046/j.1365-2958.2003.03361.x

[58] Spiers AJ, Bohannon J, Gehrig SM, Rainey PB (2003) Biofilm formation at the air-liquid interface by the Pseudomonas fluorescens SBW25 wrinkly spreader requires an acetylated form of cellulose. Mol Microbiol 50: 15–27. 14507360 10.1046/j.1365-2958.2003.03670.x

[59] Winsor GL, Lam DK, Fleming L, Lo R, Whiteside MD, et al (2011) Pseudomonas Genome Database: improved comparative analysis and population genomics capability for Pseudomonas genomes. Nucleic Acids Res 39: D596–600. 20929876 10.1093/nar/gkq869 PMC3013766

[60] Kelley LA, Sternberg MJ (2009) Protein structure prediction on the Web: a case study using the Phyre server. Nat Protoc 4: 363–371. 19247286 10.1038/nprot.2009.2

[61] Lee JE, Cornell KA, Riscoe MK, Howell PL (2001) Structure of E. coli 5′-methylthioadenosine/S-adenosylhomocysteine nucleosidase reveals similarity to the purine nucleoside phosphorylases. Structure 9: 941–953. 11591349 10.1016/s0969-2126(01)00656-6

[62] Otwinowski Z, Minor W (1997) Processing of X-ray diffraction data collected in oscillation mode. New Jersey: Elsevier. pp. 307–326. 10.1016/S0076-6879(97)76066-X 27754618

[63] Terwilliger TC, Berendzen J (1999) Automated MAD and MIR structure solution. Acta Crystallogr D Biol Crystallogr 55: 849–861. 10089316 10.1107/S0907444999000839 PMC2746121

[64] Adams PD, Afonine PV, Bunkoczi G, Chen VB, Davis IW, et al (2010) PHENIX: a comprehensive Python-based system for macromolecular structure solution. Acta Crystallogr D Biol Crystallogr 66: 213–221. 20124702 10.1107/S0907444909052925 PMC2815670

[65] Emsley P, Cowtan K (2004) Coot: model-building tools for molecular graphics. Acta Crystallogr D Biol Crystallogr 60: 2126–2132. 15572765 10.1107/S0907444904019158

[66] Adams PD, Afonine PV, Bunkoczi G, Chen VB, Davis IW, et al (2010) PHENIX: a comprehensive Python-based system for macromolecular structure solution. Acta Crystallogr D Biol Crystallogr 66: 213–221. 20124702 10.1107/S0907444909052925 PMC2815670

[67] Afonine PV, Mustyakimov M, Grosse-Kunstleve RW, Moriarty NW, Langan P, et al (2010) Joint X-ray and neutron refinement with phenix.refine. Acta Crystallogr D Biol Crystallogr 66: 1153–1163. 21041930 10.1107/S0907444910026582 PMC2967420

[68] Painter J, Merritt EA (2006) Optimal description of a protein structure in terms of multiple groups undergoing TLS motion. Acta Crystallogr D Biol Crystallogr 62: 439–450. 16552146 10.1107/S0907444906005270

[69] Heinig M, Frishman D (2004) STRIDE: a web server for secondary structure assignment from known atomic coordinates of proteins. Nucleic Acids Res 32: W500–502. 15215436 10.1093/nar/gkh429 PMC441567

[70] Dolinsky TJ, Czodrowski P, Li H, Nielsen JE, Jensen JH, et al (2007) PDB2PQR: expanding and upgrading automated preparation of biomolecular structures for molecular simulations. Nucleic Acids Res 35: W522–525. 17488841 10.1093/nar/gkm276 PMC1933214

[71] Ashkenazy H, Erez E, Martz E, Pupko T, Ben-Tal N (2010) ConSurf 2010: calculating evolutionary conservation in sequence and structure of proteins and nucleic acids. Nucleic Acids Res 38: W529–533. 20478830 10.1093/nar/gkq399 PMC2896094

[72] Krissinel E, Henrick K (2007) Inference of macromolecular assemblies from crystalline state. J Mol Biol 372: 774–797. 17681537 10.1016/j.jmb.2007.05.022

[73] Altschul SF, Gish W, Miller W, Myers EW, Lipman DJ (1990) Basic local alignment search tool. J Mol Biol 215: 403–410. 2231712 10.1016/S0022-2836(05)80360-2

[74] Edgar RC (2004) MUSCLE: multiple sequence alignment with high accuracy and high throughput. Nucleic Acids Res 32: 1792–1797. 15034147 10.1093/nar/gkh340 PMC390337

[75] Edgar RC (2004) MUSCLE: a multiple sequence alignment method with reduced time and space complexity. BMC Bioinformatics 5: 113. 15318951 10.1186/1471-2105-5-113 PMC517706

[76] Zdanov A, Li Y, Bundle DR, Deng SJ, MacKenzie CR, et al (1994) Structure of a single-chain antibody variable domain (Fv) fragment complexed with a carbohydrate antigen at 1.7-A resolution. Proc Natl Acad Sci U S A 91: 6423–6427. 7517550 10.1073/pnas.91.14.6423 PMC44214

[77] Walvoort MT, Volbeda AG, Reintjens NR, van den Elst H, Plante OJ, et al (2012) Automated Solid-Phase Synthesis of Hyaluronan Oligosaccharides. Org Lett 14: 3776–3779. 22780913 10.1021/ol301666n

[78] Walvoort MT, van den Elst H, Plante OJ, Krock L, Seeberger PH, et al (2012) Automated solid-phase synthesis of beta-mannuronic acid alginates. Angew Chem Int Ed Engl 51: 4393–4396. 22334421 10.1002/anie.201108744

[79] Lin H, Kitova EN, Klassen JS (2013) Quantifying protein-ligand interactions by direct electrospray ionization-MS analysis: evidence of nonuniform response factors induced by high molecular weight molecules and complexes. Anal Chem 85: 8919–8922. 24044528 10.1021/ac401936x

[80] El-Hawiet A, Kitova EN, Klassen JS (2012) Quantifying carbohydrate-protein interactions by electrospray ionization mass spectrometry analysis. Biochemistry 51: 4244–4253. 22564104 10.1021/bi300436x

[81] Sun J, Kitova EN, Wang W, Klassen JS (2006) Method for distinguishing specific from nonspecific protein-ligand complexes in nanoelectrospray ionization mass spectrometry. Anal Chem 78: 3010–3018. 16642987 10.1021/ac0522005

[82] Chen VB, Arendall WB 3rd, Headd JJ, Keedy DA, Immormino RM, et al (2010) MolProbity: all-atom structure validation for macromolecular crystallography. Acta Crystallogr D Biol Crystallogr 66: 12–21. 20057044 10.1107/S0907444909042073 PMC2803126

