# Supplementary material for: P. aeruginosa SGNH Hydrolase-Like Proteins AlgJ and AlgX Have Similar Topology but Separate and Distinct Roles in Alginate Acetylation
Source: PLoS Pathog. 2014 Aug 28;10(8):e1004334. doi: 10.1371/journal.ppat.1004334 (PMC4148444; doi:10.1371/journal.ppat.1004334)
Supplement: Table S1 — Bacterial strains and plasmids used in this study. (DOCX) [file ppat.1004334.s001.docx]

**Supplementary Information**

**Table S1: Bacterial Strains and Plasmids used in this Study**.

Antibiotic resistance marker is abbreviated as follows: Kan^R^, kanamycin resistance. The restriction sites *Nde*I and *Xho*I are underlined.

| **Strain or plasmid** | **Characteristics** | **Source** |
| --- | --- | --- |
| **Strain** |  |  |
| ***E. coli*** |  |  |
| *E. coli* TOP10^TM^ | F^-^ *mcr*A Δ(*mrr-hsd*RMS-*mcr*BC) φ80*lac*ZΔM15 Δ*lac*X74 *rec*A1 *ara*D139 Δ(*ara-leu*) 7697 *gal*U *gal*K *rps*L (Str^r^) *end*A1 *nup*G λ- | Invitrogen |
| *E. coli* BL21-CodonPlus^®^ (DE3)-RP | F^-^ *omp*T *hsd*S(r_B_^-^ m_B_^-^) *dcm*^+^ Tet^r^ *gal* λ(DE3) *end*A [*arg*U *pro*L Cam^r^] | Stratagene |
| B834(DE3) | F^-^ *omp*T *hsd*SB(r_B_^-^ m_B_^-^) *gal* *dcm* *met*(DE3) | Novagen |
| **Plasmid** |  |  |
| pPLHPaAlgJ | pET28a(+) with *Pa*AlgJ_79-379_ with N-terminal 6xHis tag, Kan^R^, *lacI* | This study |
| pPLHPaAlgJ-193 | pPLHPaAlgJ (D193A) | This study |
| pPLHPaAlgJ-195 | pPLHPaAlgJ (H195A) | This study |
| pPLHPaAlgJ-297 | pPLHPaAlgJ (S297A) | This study |
| pPLHPpAlgJ1^*^ | pET28a(+) with *Pa*AlgJ_79-379_ with N-terminal 6xHis-tag, Kan^R^, *lacI* | This study |
| pPLHPpAlgJ2^**^ | pET28a(+) with *Pa*AlgJ_79-379_ with N-terminal and C-terminal 6xHis-tag, Kan^R^, *lacI* | This study |
| pPLHPpAlgJ1-190 | pPLHPpAlgJ1 (D190A) | This study |
| pPLHPpAlgJ1-192 | pPLHPpAlgJ1 (H192A) | This study |
| pPLHPpAlgJ1-288 | pPLHPpAlgJ1 (S288A) | This study |
| **Primer name** | **Sequence (5’ – 3’)** |  |
| PaAlgJFOR | GTTCATATGCACGAAGGCCGCCCCGGCGT  GTTCTCGAGTCAGCCGCTGGCCTTCAGCTGG  TTCCTGCGCACCGCCACCCACTGGTCGCCG  CGGCGACCAGTGGGTGGCGGTGCGCAGGAA  CGCACCGACACCGCCTGGTCGCCGCTCGGC  GCCGAGCGGCGACCAGGCGGTGTCGGTGCG  CTGGTCGGCACCGCCTACAGCGCCAACCCC  GGGGTTGGCGCTGTAGGCGGTGCCGACCAG | |
| PaAlgJREV |  |  |
| PaAlgJD193AFOR |  |  |
| PaAlgJD193AREV |  |  |
| PaAlgJH195AFOR |  |  |
| PaAlgJH195AREV |  |  |
| PaAlgJS297AFOR |  |  |
| PaAlgJS297AREV |  |  |
| PpAlgJFOR2^*^ | GTTCATATGAACGAAGGCCGCCCAGGTGT  GTTCTCGAGACGGGAGTTTTTCAGCTGGG  GTTCATATGAACGAAGGCCGCCCAGGTGT  GTTCTCGAGTCAACGGGAGTTTTTCAGCT  TTCCTGCGTACCGCTACCCACTGGACGCCT  AGGCGTCCAGTGGGTAGCGGTACGCAGGAA  CGTACCGATACCGCCTGGACGCCTATGGGT  ACCCATAGGCGTCCAGGCGGTATCGGTACG  CTGGTCGGCACCGCCTACAGCGCCAACCCC  GGGGTTGGCGCTGTAGGCGGTGCCGACCAG | |
| PpAlgJREV2^*^ |  |  |
| PpAlgJFOR1^**^ |  |  |
| PpAlgJREV1^**^ |  |  |
| PpAlgJD190AFOR |  |  |
| PpAlgJD190AREV |  |  |
| PpAlgJH192AFOR |  |  |
| PpAlgJH192AREV |  |  |
| PpAlgJS288AFOR |  |  |
| PpAlgJS288AREV |  |  |

^*^ This construct contained both N- and C-terminal His_6_ tags and was used for crystallization.

^**^ This construct only contained the N-terminal His_6_ tag and was used for functional characterization.
